# Beyond homogeneity: celebrity worship types and perceived stress as key roles in the relationship between smartphone addiction and cyberbullying among young women

**DOI:** 10.3389/fpsyg.2026.1878172

**Published:** 2026-08-20

**Authors:** Mengru Sun

**Affiliations:** 1 College of Media and International Culture, Zhejiang University, Hangzhou, China; 2 International Communication Institute, Zhejiang University, Hangzhou, China

**Keywords:** celebrity worship, cyberbullying penetration, perceived stress, smartphone addiction, women, young adults

## Abstract

**Background:**

The rapid proliferation of information and communication technologies (ICTs) has given rise to increasingly sophisticated forms of cyberbullying, with expanding penetration scope and escalating severity of harm. Despite well-documented gender differences in cyberbullying involvement, the underlying mechanisms among young female adults remain insufficiently understood.

**Methods:**

The present study examined the relationship between smartphone addiction and cyberbullying penetration among young female adults, investigating the mediating role of celebrity worship (Entertainment-Social, Intense-Personal, and Borderline Pathological dimensions) and the moderating role of perceived stress. A moderated mediation model was proposed and empirically tested using survey data from 524 female undergraduate students in eastern China.

**Results:**

Results indicated that smartphone addiction was positively associated with cyberbullying penetration both directly and indirectly. Specifically, the direct effect of smartphone addiction on cyberbullying penetration was significant only when Entertainment-Social worship served as the mediator. Intense-Personal worship and Borderline Pathological worship significantly mediated the smartphone addiction–cyberbullying penetration relationship, whereas Entertainment-Social worship did not. Furthermore, perceived stress significantly moderated the association between Borderline Pathological worship and cyberbullying penetration, but did not moderate the pathways involving Entertainment-Social or Intense-Personal worship.

**Discussion:**

The findings contribute to the growing literature on the adverse behavioral consequences of smartphone addiction, particularly its association with increased cyberbullying involvement. Moreover, this study advances understanding of the gendered mechanisms underlying cyberbullying by focusing on young women. We also highlight the complex, multi-determined nature of cyberbullying and reinforce the necessity of examining both risk and protective factors within integrated theoretical frameworks that account for individual, relational, and contextual influences.

## Introduction

The rapid advancement of information and communication technologies (ICTs) has fundamentally transformed interpersonal communication among young adults, with 96% of individuals aged 18 to 29 owning a smartphone and 84% actively using social media (Pew Research Center, 2022; Valenzuela et al., 2009). While these technologies have facilitated unprecedented connectivity, they have simultaneously engendered new forms of interpersonal violence, most notably cyberbullying penetration (Aizenkot and Kashy-Rosenbaum, 2021). Cyberbullying, defined as repeated aggressive behavior perpetrated through electronic means with the intent to harm a less powerful individual (Gaffney et al., 2019), represents a digital extension of traditional bullying that has become increasingly prevalent and severe in scope.

Epidemiological evidence indicates that cyberbullying has become a predominant form of aggression among young adults, with cross-national prevalence rates ranging from 20 to 40% and rates of approximately 10–15% among college students specifically (Aboujaoude et al., 2015; Selkie et al., 2015). The incidence of cyberbullying appears to increase from early adolescence through adulthood, with Canada (23.8%) and China (23.0%) reporting the highest rates globally (Aboujaoude et al., 2015; Brochado et al., 2017). The antecedents of cyberbullying is multifactorial, encompassing individual characteristics, peer dynamics, family functioning, school climate, and broader sociocultural influences (Slonje et al., 2013; Shahzad et al., 2026).

Cyberbullying penetration involves the use of electronic communication to threaten, harass, humiliate, or socially exclude others (Langos, 2012). Several features distinguish cyberbullying from its traditional counterpart and may amplify its harmful effects. The anonymity afforded by online platforms reduces perpetrators’ accountability and facilitates behavioral disinhibition (Slonje et al., 2013). Unlike traditional bullying, where victims can temporarily escape harassment in physical settings, cyberbullying transcends temporal and spatial boundaries, subjecting victims to persistent victimization. Moreover, the viral nature of digital content enables rapid dissemination to potentially unlimited audiences (Squicciarini et al., 2015), while the absence of face-to-face interaction prevents perpetrators from perceiving victims’ emotional distress, potentially escalating the severity of aggressive acts. Critically, whereas school-based bullying is subject to institutional surveillance and intervention, online aggression operates with minimal regulatory oversight (Campbell et al., 2012).

Given these distinctive features, investigating the antecedents of cyberbullying among young adults is both timely and imperative. Young female adults constitute a particularly vulnerable population in this context. Women use social networking sites at substantially higher rates (77%) than men (61%), and these platforms represent the primary venues for cyberbullying perpetration (Dredge et al., 2014; Pew Research Center, 2021). Consistent with this differential exposure, the majority of research indicates that women are disproportionately involved in cyberbullying both as victims and as perpetrators (Heiman and Olenik-Shemesh, 2015; Selkie et al., 2015).

**Figure 1:**
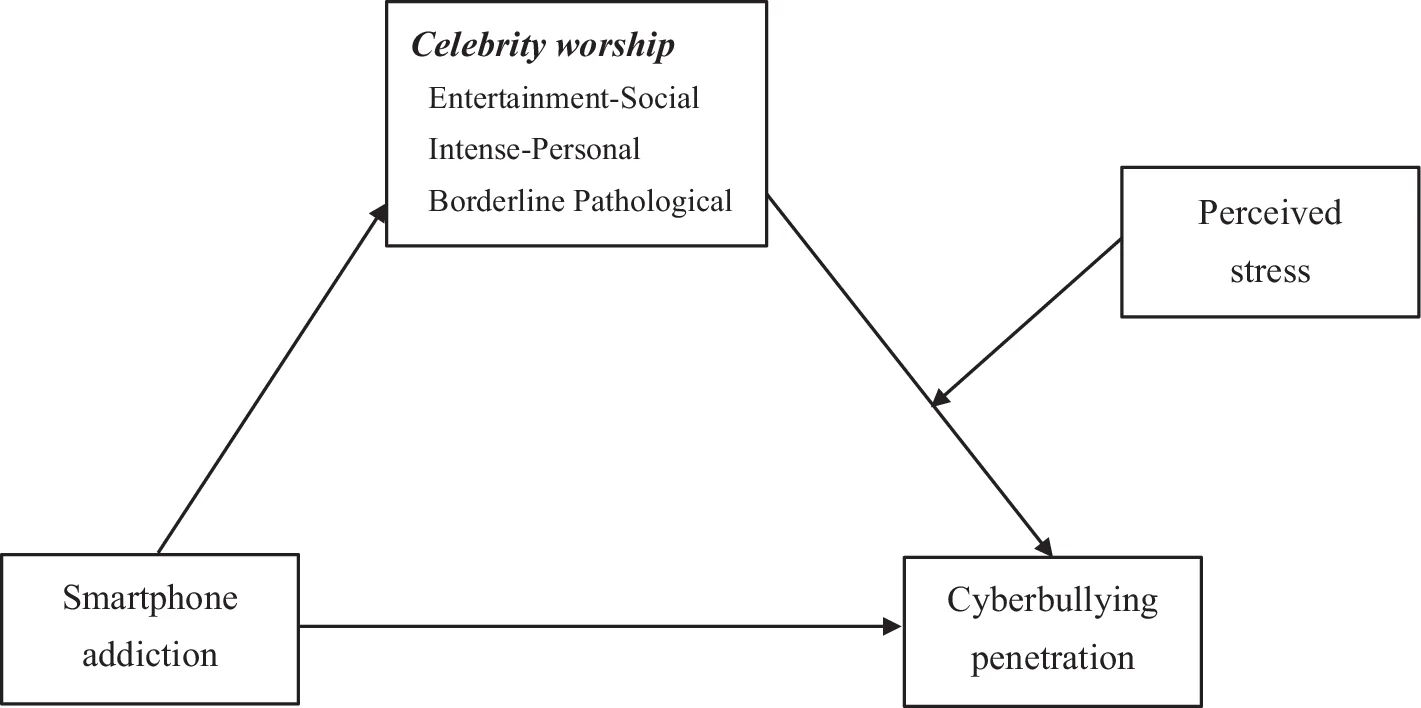
The hypothesized model in the present study.

Despite these pronounced gender differences, the mechanisms underlying cyberbullying among young female adults remain poorly understood. Although smartphone addiction and celebrity worship have each received considerable scholarly attention, few empirical studies have examined how smartphone addiction influences cyberbullying penetration through the mechanism of celebrity worship (Ouvrein et al., 2017). To address this gap, the present study proposes and tests a moderated mediation model examining the interplay among smartphone addiction, celebrity worship, perceived stress, and cyberbullying penetration in a sample of young female adults. Specifically, we hypothesize that smartphone addiction influences cyberbullying penetration both directly and indirectly through celebrity worship, with perceived stress moderating the association between celebrity worship and cyberbullying penetration (see Figure 1).

## Literature review

### Smartphone addiction and cyberbullying

Smartphone addiction, also termed “smartphone dependence,” has been conceptualized in multiple ways across the literature. Some scholars characterize it as a chronic or periodic obsessive state arising from repeated smartphone use, marked by a persistent and compelling sense of psychological dependence (e.g., Wang and Lee, 2020). Others draw parallels with behavioral addiction in clinical psychology, defining it as a ritualistic, habitual physiological and psychological state involving problematic and pathological use patterns (e.g., Van Deursen et al., 2015). Synthesizing these perspectives, smartphone addiction encompasses not merely a behavioral pattern but also a psychological state of compulsive over-reliance stemming from excessive smartphone engagement (Al-Barashdi et al., 2015).

Smartphones have become integral to communication, mobile payment, education, and entertainment in the daily lives of young adults. However, this developmental period is characterized by incomplete personality maturation, unstable self-control, and still-developing self-regulatory capacities. Excessive smartphone reliance during this vulnerable period may produce pronounced negative consequences for social adjustment (Al-Kandari and Al-Sejari, 2021). In the domain of interpersonal relationships, smartphone addiction has been associated with heightened feelings of alienation and loneliness among college students, and the concomitant deficits in social skills and social support may precipitate interpersonal maladjustment. Importantly, accumulating evidence indicates that smartphone addiction constitutes a risk factor for cyberbullying perpetration (Fabito et al., 2018; Gül et al., 2019; Tsimtsiou et al., 2018; Vandebosch and Van Cleemput, 2009; Woo et al., 2018). Cyberbullying encompasses a range of aggressive behaviors perpetrated through electronic means, including verbal abuse, rumor spreading, incitement, threats, non-consensual sharing of private images or videos, and deliberate exclusion from online communities (Catone et al., 2020; Mesch, 2009).

Several mechanisms may account for the association between smartphone addiction and cyberbullying. First, with respect to social cognition, young adults are in a transitional developmental stage requiring the formation of coherent self-concepts and understanding of the external world, processes integral to successful socialization (Lee and Seo, 2014). However, individuals with smartphone addiction may encounter difficulties in cognitive development, as their reliance on smartphones for information acquisition reduces opportunities for knowledge construction through active reflection and practice. Consequently, they may erroneously perceive cyberbullying as a normative online behavior prevalent among peers (Fabito et al., 2018; Tsimtsiou et al., 2018; Woo et al., 2018).

Second, smartphone addiction appears to erode adherence to social norms (Hong et al., 2021). The privacy afforded by mobile networks enables individuals to disseminate information without concern for social detection. This perceived anonymity reduces the opportunity cost of deviant behavior and, under conditions of diffused responsibility, further compromises self-regulatory capacity, thereby increasing the likelihood of norm-violating behaviors such as cyberbullying during the socialization process.

Third, and perhaps most critically, smartphone addiction immerses young adults in virtual environments at the expense of real-world social engagement, thereby impeding healthy personality development (Mahapatra, 2019). A central developmental task during young adulthood is the formation of a coherent self-identity through social education and cultural internalization. The derealization and personality distortion associated with excessive smartphone use are antithetical to this developmental process. Based on the foregoing theoretical analysis, we propose the following hypothesis:

*Hypothesis 1*: Smartphone addiction is positively associated with cyberbullying penetration.

### Celebrity worship and cyberbullying

ICTs have simultaneously enriched the leisure lives of young adults and created new avenues for celebrity engagement. On one hand, digital platforms provide novel channels for idol worship, intensifying parasocial interactions and fulfilling psychological needs for connection and identification (Duffy and Pooley, 2019). Consistent with this, problematic Internet use has been associated with elevated levels of celebrity worship (Zsila et al., 2018). On the other hand, online fan communities have proliferated rapidly, and this form of digital worship is increasingly shaping the online behaviors of young adults (Yan and Yang, 2021). With the diversification of entertainment formats, celebrity worship has transcended traditional face-to-face interaction and shifted toward Internet-mediated online engagement. Research suggests that the enhanced accessibility of celebrities through digital platforms may foster extreme addictive attachments, resulting in irrational and maladaptive behavioral outcomes (Yan and Yang, 2021).

Celebrity worship is defined as an individual’s identification with and emotional attachment to a favored figure, encompassing both affective projection and behavioral tendencies directed toward the object of admiration (McCutcheon et al., 2002). This phenomenon is particularly salient during adolescence and young adulthood, a period characterized by heightened self-awareness and the developmental transition from proximal attachment figures to distal idealized objects (Häkkänen-Nyholm, 2021). In this process, celebrities may supplant parents as primary objects of emotional dependence (Maltby et al., 2004). According to the absorption-addiction model, celebrity worship originates from psychological absorption with and infatuation toward a celebrity, whereby individuals with fragile identity structures and deficient meaningful relationships attempt to psychologically “absorb” the celebrity to compensate for identity deficits (McCutcheon et al., 2002). The Celebrity Attitude Scale delineates three progressive dimensions: Entertainment-Social worship, characterized by information-seeking and social discussion about celebrities; Intense-Personal worship, involving obsessive preoccupation with a celebrity’s personal life; and Borderline Pathological worship, reflecting extreme behavioral tendencies including willingness to engage in unlawful acts at a celebrity’s behest (McCutcheon et al., 2002). These dimensions represent a continuum from normative admiration to pathological fixation.

As a projection of the ideal self, celebrity worship may confer certain adaptive functions, including facilitating self-identity formation and enhancing self-concept clarity (Maltby et al., 2006). However, with the advent of online worship, the maladaptive consequences have become increasingly apparent. Baek et al. (2013) characterized the individual–celebrity relationship as parasocial in nature, unidirectional, lacking reciprocal interaction, and positively associated with loneliness and interpersonal distrust as individuals progressively disengage from offline social contexts (Shabahang et al., 2020).

Online fan communities, while fostering collective identity among admirers of the same celebrity, may simultaneously facilitate maladaptive online behaviors. The social identity derived from group membership can attenuate individual sense of social responsibility, enabling members to rationalize unethical conduct through moral disengagement mechanisms and weakening the restraining influence of moral norms. For instance, inter-fandom conflicts have rendered online verbal aggression increasingly commonplace among young adults. Regarding gender differences, celebrity worship is more prevalent among women, potentially attributable to greater tendencies toward romantic fantasizing about idols and heightened susceptibility to group pressure. Women demonstrate greater proneness to romantic parasocial attachment, making them more likely to envision celebrities as romantic partners (Jung and Hwang, 2016; Maltby et al., 2005). Furthermore, gender differences exist in the types of celebrities worshiped: women tend to favor singers and film stars, whereas men gravitate toward politicians and other status-oriented figures (Zsila et al., 2018). Zsila et al. (2021) attributed this divergence to gender role socialization, whereby men prioritize status-signaling attributes such as wealth and reputation, while women place greater emphasis on physical appearance. Based on the foregoing analysis, we propose the following hypothesis:

*Hypothesis 2*: Celebrity worship mediates the relationship between smartphone addiction and cyberbullying penetration.

### Perceived stress and cyberbullying

General strain theory (GST) posits that individuals who experience negative stimuli or fail to achieve positively valued goals develop negative affective states, such as anger, frustration, and depression, that increase the propensity for criminal or deviant behavior as a maladaptive coping response (Agnew, 1999; Broidy and Agnew, 1997). When young adults are subjected to multiple concurrent stressors (e.g., academic difficulties, perceived injustice, family financial strain, school transitions, and interpersonal conflicts), the resultant negative emotions may precipitate aggressive or delinquent behaviors. Hinduja (2007) proposed that individuals under strain adopt compensatory strategies to alleviate negative affect. For young adults, bullying, whether traditional or cyber, may function as a maladaptive regulatory strategy that temporarily restores a sense of agency and control.

Consistent with this theoretical framework, cyberbullying may serve as an emotion-regulation strategy through which young adults release accumulated stress and negative affect, deriving a sense of dominance and gratification from harming peers (Hay et al., 2010). Paez (2018) demonstrated that both personal and social stressors derived from GST were positively associated with cyberbullying penetration among young adults. For instance, individuals with low peer acceptance and negative school attitudes exhibited greater cyberbullying involvement than those with high peer acceptance and positive school orientations. Additional studies have corroborated the link between perceived stress and increased engagement in violent and bullying behaviors (Patchin and Hinduja, 2011). Extending this logic to the present model, we hypothesized that the positive association between celebrity worship and cyberbullying penetration would be amplified under conditions of elevated perceived stress among young female adults. Accordingly, we propose the following hypothesis:

*Hypothesis 3*: Perceived stress positively moderates the relationship between celebrity worship and cyberbullying penetration.

## Method

### Participants and procedures

Data were collected from 563 participants as part of a broader survey on youth social development. The present study utilized data pertaining to smartphone use, celebrity worship, and cyberbullying. Female undergraduate students from a university in eastern China participated voluntarily in exchange for a small number of additional academic credits. Participants completed an online survey administered through Sojump (Wenjuanxing), the largest online survey platform in China. After screening for response validity and excluding participants under 18 years of age, the final analytic sample comprised 524 participants.

All 524 participants were young female adults and undergraduate students. The majority identified as Han ethnicity. Regarding residential background, 51.5% were urban residents (*n* = 270) and 48.5% were rural residents (*n* = 254). Participants ranged in age from 18 to 26 years (*M* = 19.94, SD = 1.17). The majority reported moderate academic performance (*n* = 303, 57.8%) and moderate household socioeconomic status (*n* = 369, 70.4%). The mean Body Mass Index (BMI) was 20.48 (SD = 3.77).

### Measures

#### Smartphone addiction

Smartphone addiction was assessed using the scale developed by Kwon et al. (2013). A representative item was “I frequently check my phone when talking with friends and family.” Responses were recorded on a 5-point Likert scale (1 = completely disagree, 5 = completely agree). In the present sample, *M* = 2.83, SD = 0.77, Cronbach’s *α* = 0.73.

#### Celebrity worship

Celebrity worship was measured using the scale adapted from McCutcheon et al. (2002), comprising three dimensions: Entertainment-Social, Intense-Personal, and Borderline Pathological worship. All items were rated on a 5-point Likert scale (1 = completely disagree, 5 = completely agree). A representative item for Entertainment-Social worship was “My friends and I enjoy discussing what my favorite celebrity has done” (*M* = 2.94, SD = 0.76, Cronbach’s *α* = 0.71). A representative item for Intense-Personal worship was “I am fascinated by the personal details of my favorite celebrity’s life” (*M* = 2.64, SD = 0.89, Cronbach’s *α* = 0.81). A representative item for Borderline Pathological worship was “If I were lucky enough to meet my favorite celebrity and he/she asked me to do something illegal, I might do it” (*M* = 1.98, SD = 0.73, Cronbach’s *α* = 0.68).

#### Perceived stress

Perceived stress was assessed using the scale adapted from Ye et al. (2020). A representative item was “I worry about drifting apart from my classmates and friends.” Responses were recorded on a 5-point scale (1 = not at all stressed, 5 = very stressed). In the present sample, *M* = 2.67, SD = 0.72, Cronbach’s *α* = 0.76.

#### Cyberbullying penetration

Cyberbullying penetration was measured using the scale adapted from Wright and Li (2013). A representative item was “I insult other classmates or friends online or via text messages.” Responses were recorded on a 5-point scale (1 = never, 5 = always). In the present sample, *M* = 1.41, SD = 0.63, Cronbach’s *α* = 0.90.

#### Control variables

Demographic variables including ethnicity, residential region, academic performance, and family socioeconomic status were controlled. Additionally, BMI was included as a covariate given its documented association with behavioral outcomes among young female adults (Mamun et al., 2013). These covariates were incorporated to mitigate potential confounding effects.

### Analysis

Data were processed using SPSS 25.0. Missing data were handled through mean imputation, whereby missing values were replaced with the mean score of the corresponding item. Descriptive statistics, independent-samples t-tests, and bivariate correlation analyses were conducted to characterize the sample and examine preliminary associations. Bivariate correlations indicated that smartphone addiction was positively associated with Intense-Personal worship, Borderline Pathological worship, perceived stress, and cyberbullying penetration. Mediation and moderated mediation effects were tested using Hayes’ (2018) PROCESS macro. Specifically, Model 4 was employed for simple mediation analyses, and Model 14 was used for moderated mediation analyses. All indirect effects were estimated using 5,000 bootstrap samples with 95% bias-corrected confidence intervals.

## Results

### Descriptive statistics and correlations

Bivariate correlation analyses revealed that academic performance was positively associated with Entertainment-Social worship (*r* = 0.09, *p* < 0.05), and BMI was positively associated with cyberbullying penetration (*r* = 0.09, *p* < 0.05). No other demographic variables were significantly correlated with the study variables (see Table 1).

**Table 1:** Bivariate correlations among the variables in the present study (*N* = 525).

Variables	1	2	3	4	5	6	7	8	9	10	11
1. Ethnicity	–										
2. Residence locale	−0.00	–									
3. Academic performance	−0.12**	0.02	–								
4. Family socioeconomic status	−0.09*	−0.16***	0.24***	–							
5. BMI	0.04	0.04	−0.05	−0.02	–						
6. Smartphone addiction	−0.02	0.03	−0.05	−0.02	0.02	–					
7. Entertainment-social worship	0.00	0.04	0.09*	0.07	−0.04	0.09	–				
8. Intense-personal worship	−0.08	0.00	0.03	0.11	0.02	0.09*	0.70***	–			
9. Borderline pathological worship	−0.03	−0.04	0.03	0.08	0.04	0.09*	0.46***	0.64***	–		
10. Perceived stress	−0.04	0.08	−0.01	−0.04	0.00	0.10*	0.09*	0.09*	0.08*	–	
11. Cyberbullying penetration	0.06	−0.06	−0.01	0.00	0.09*	0.09*	0.10*	0.15***	0.28***	0.15***	–

*p < 0.05, **p < 0.01, ***p < 0.001.

### Hypothesis testing

To test the hypothesized mediation and moderated mediation effects, parallel analyses were conducted across the three dimensions of celebrity worship. First, simple mediation analyses were performed using PROCESS Model 4 (Hayes, 2018), estimating both indirect effects (smartphone addiction → celebrity worship → cyberbullying penetration) and direct effects (smartphone addiction → cyberbullying penetration). Three separate mediation models were estimated (see Tables 2–4). Subsequently, moderated mediation analyses were conducted using PROCESS Model 14, incorporating perceived stress as a moderator of the celebrity worship → cyberbullying penetration pathway. All analyses employed 5,000 bootstrap samples with 95% bias-corrected confidence intervals. The comparative results across the three models are presented in Table 5.

**Table 2:** The mediation effect of entertainment-social worship in the relationship between smartphone addiction and cyberbullying penetration.

Predictors	Model 1 (entertainment-social worship)			Model 2 (cyberbullying penetration)		
	*b*	*t*	95% CI	*b*	*t*	95% CI
Ethnicity	0.03	0.79	[−0.14, 0.33]			
Residence locale	0.05	1.14	[−0.05, 0.21]			
Academic performance	0.06	1.37	[−0.03, 0.16]			
Family socioeconomic status	0.05	1.12	[−0.05, 0.17]			
BMI	−0.04	−0.95	[−0.03, 0.01]			
Smartphone addiction	0.11*	2.41	[0.02, 0.19]			
Entertainment-Social worship				0.09*	2.13	[0.01, 0.15]
*R* ^2^		0.02			0.03	
*F*		1.97			2.53	

*p < 0.05, **p < 0.01, ***p < 0.001.

**Table 3:** Mediation effect of intense-personal worship in the relationship between smartphone addiction and cyberbullying penetration.

Predictors	Model 1 (intense-personal worship)			Model 2 (cyberbullying penetration)		
	*b*	*t*	95% CI	*b*	*t*	95% CI
Ethnicity	−0.06	1.58	[−0.47, 0.35]			
Residence locale	0.01	0.30	[−0.13, 0.18]			
Academic performance	−0.00	−0.02	[−0.11, 0.11]			
Family socioeconomic status	0.05	1.19	[−0.05, 0.22]			
BMI	0.02	0.46	[−0.02, 0.03]			
Smartphone addiction	0.10*	2.36	[0.02, 0.22]			
Intense-personal celebrity worship				0.14**	3.27	[0.04, 0.16]
*R* ^2^		0.02			0.05	3.43
*F*		1.62			3.44	

*p < 0.05, **p < 0.01, ***p < 0.001.

**Table 4:** Mediation effect of borderline pathological worship in the relationship between smartphone addiction and cyberbullying penetration.

Predictors	Model 1 (borderline pathological worship)			Model 2 (cyberbullying penetration)		
	*b*	*t*	95% CI	*b*	*t*	95% CI
Ethnicity	−0.01	−0.21	[−0.25, 0.20]			
Residence locale	−0.03	−0.65	[−0.17, 0.08]			
Academic performance	0.01	0.19	[−0.08, 0.10]			
Family socioeconomic status	0.05	0.99	[−0.05, 0.16]			
BMI	0.04	0.82	[−0.01, 0.02]			
Smartphone addiction	0.11*	2.43	[0.02, 0.18]			
Borderline pathological celebrity worship				0.27***	6.32	[0.16, 0.31]
*R* ^2^		0.02			0.10	
*F*		1.40			7.74	

*p < 0.05, **p < 0.01, ***p < 0.001.

**Table 5:** Mediation analyses using celebrity worship as the mediator and moderation analysis using perceived stress as the moderator.

Path	Entertainment-social worship model		Intense-personal worship model		Borderline pathological worship model	
	Effect	95% CI	Effect	95% CI	Effect	95% CI
Direct effect						
Smartphone addiction → cyberbullying penetration	0.07*	[0.00, 0.14]	0.06	[−0.00, 0.14]	0.06	[−0.01, 0.12]
Indirect effect						
Smartphone addiction → celebrity worship→ cyberbullying penetration	0.01	[−0.00, 0.03]	0.01*	[0.00, 0.03]	0.03*	[0.00, 0.06]
Moderation effect						
WeRun use × perceived stress (on cyberbullying penetration)	0.05	[−0.04, 0.14]	0.04	[−0.03, 0.11]	0.15***	[0.06, 0.24]

Covariates: demographic variables (ethnicity, residence locale, academic performance, family socioeconomic status, and BMI). *p < 0.05, **p < 0.01, ***p < 0.001.

Regarding Hypothesis 1, smartphone addiction was directly and positively associated with cyberbullying penetration when Entertainment-Social worship served as the mediator [*b* = 0.07, *t* = 1.98, 95% CI (0.00, 0.14)]. However, the direct effect was non-significant when Intense-Personal worship [*b* = 0.06, *t* = 1.88, 95% CI (−0.00, 0.14)] or Borderline Pathological worship [*b* = 0.06, *t* = 1.60, 95% CI (−0.01, 0.12)] served as the mediator. Thus, Hypothesis 1 was partially supported.

Regarding Hypothesis 2, the indirect effects of smartphone addiction on cyberbullying penetration through Intense-Personal worship [*b* = 0.01, 95% CI (0.00, 0.03)] and Borderline Pathological worship (*b* = 0.03, 95% CI [0.00, 0.06]) were statistically significant, as evidenced by confidence intervals excluding zero. In contrast, the indirect effect through Entertainment-Social worship was non-significant [*b* = 0.01, 95% CI (−0.00, 0.03)]. Thus, Hypothesis 2 was partially supported.

Regarding Hypothesis 3, the interaction between perceived stress and Entertainment-Social worship in predicting cyberbullying penetration was non-significant [*b* = 0.05, *t* = 1.05, 95% CI (−0.04, 0.14)], as was the interaction between perceived stress and Intense-Personal worship [*b* = 0.04, t = 1.10, 95% CI (−0.03, 0.11)]. However, perceived stress significantly moderated the association between Borderline Pathological worship and cyberbullying penetration [*b* = 0.15, *t* = 3.38, 95% CI (0.06, 0.24)], indicating that the positive relationship between Borderline Pathological worship and cyberbullying penetration was strengthened under conditions of elevated perceived stress. Thus, Hypothesis 3 was partially supported.

## Discussion

The present study proposed and empirically tested a moderated mediation model of cyberbullying penetration among young female adults, examining the mediating role of celebrity worship in the smartphone addiction–cyberbullying penetration link and the moderating role of perceived stress. The findings contribute to the growing literature on the adverse behavioral consequences of smartphone addiction, particularly its association with increased cyberbullying involvement. Moreover, this study advances understanding of the gendered mechanisms underlying cyberbullying by focusing on young women, a population that, despite being disproportionately involved in cyberbullying, has received comparatively limited empirical attention.

The first key finding was that smartphone addiction was positively associated with cyberbullying penetration, consistent with prior research demonstrating that problematic smartphone use predicts cyberbullying perpetration among college students (Qudah et al., 2019) and that the psychological consequences of smartphone addiction, including depression, withdrawal, impaired impulse control, and anxiety, are associated with aggressive online behaviors (Chiu et al., 2013). Although direct evidence linking smartphone addiction specifically to cyberbullying remains limited, a broader body of literature has established a positive association between Internet addiction and cyberbullying (Athanasiades et al., 2015; Chang et al., 2015; Kırcaburun and Bastug, 2016; Nartgün and Cicioğlu, 2015). Notably, a cross-sectional study by AlQaderi et al. (2023) among young adults in the United Arab Emirates found that while phone addiction was positively linked to anxiety and depression, it was not directly associated with cyberbullying perpetration, suggesting that the relationship between problematic phone use and online aggression may be more nuanced than previously assumed and potentially contingent upon cultural context or intervening mechanisms. The present findings extend this literature by demonstrating that the direct effect of smartphone addiction on cyberbullying penetration was conditional upon the type of celebrity worship serving as the mediator. Specifically, the direct effect was significant only when Entertainment-Social worship was the mediator, whereas it became non-significant when Intense-Personal or Borderline Pathological worship served as mediators. Drawing on uses and gratifications theory, this pattern suggests that different motivational underpinnings of smartphone use may differentially influence cyberbullying behavior. Collectively, these findings indicate that the smartphone addiction–cyberbullying association may be relatively attenuated and largely operating through indirect pathways, underscoring the need for future research to identify additional mediating mechanisms and to differentiate among subtypes of problematic smartphone use.

The second key finding pertains to the mediating role of celebrity worship. Both Intense-Personal and Borderline Pathological worship significantly mediated the smartphone addiction–cyberbullying penetration link, whereas Entertainment-Social worship did not. While prior research has established a positive association between problematic Internet use and celebrity worship (Zsila et al., 2018), the differential effects of specific worship dimensions on cyberbullying have received insufficient attention (Li et al., 2018). A recent study by Li et al. (2025) further illuminates the psychosocial antecedents of celebrity worship, demonstrating that maternal overprotection significantly predicts idol worship among college students through a chain mediating pathway involving reactive anger and cybervictimization. This finding underscores the developmental roots of celebrity worship and suggests that early family dynamics may indirectly shape online aggressive behaviors through worship-related mechanisms. In the present study, smartphone addiction was positively associated with Intense-Personal and Borderline Pathological worship but not with Entertainment-Social worship. This pattern is theoretically coherent: smartphone addiction, as a problematic behavioral pattern, is more likely to be associated with the more pathological dimensions of celebrity worship rather than the relatively benign Entertainment-Social dimension. Furthermore, only the more pathological worship dimensions (Intense-Personal and Borderline Pathological) were associated with subsequent cyberbullying, while Entertainment-Social worship was not. This dissociation suggests that Entertainment-Social worship may retain adaptive social functions, whereas the obsessive and boundary-dissolving characteristics of Intense-Personal and Borderline Pathological worship may erode moral restraint and facilitate aggressive online behaviors (Ouvrein et al., 2020).

The third key finding concerns the moderating role of perceived stress. Perceived stress significantly amplified the positive association between Borderline Pathological worship and cyberbullying penetration but did not moderate the pathways involving Entertainment-Social or Intense-Personal worship. This pattern is consistent with general strain theory, which posits that negative affective states arising from stressors deplete self-regulatory resources and increase the propensity for deviant coping strategies. Under conditions of elevated stress, fans may engage in cyberbullying against perceived threats to their admired celebrity’s image, or alternatively, their excessive worship may devolve into attempts to control or harass the celebrity themselves (Ouvrein et al., 2017). This interpretation is corroborated by a moderated mediation study by Wang and Jiang (2022), which demonstrated that smartphone addiction partially mediated the association between parental neglect and cyberbullying perpetration, while self-regulation moderated both the direct and indirect pathways. Their finding that individual regulatory resources critically determine whether risk factors translate into aggressive online behaviors aligns with our observation that perceived stress, as an indicator of depleted regulatory capacity, selectively intensifies the most pathological worship–cyberbullying pathway. Thus, the present findings advance understanding of the conditions under which celebrity worship translates into cyberbullying, highlighting the exacerbating role of stress-induced self-regulatory failure.

These findings should be situated within the broader context of recent advances in cyberbullying research employing mediation and moderation frameworks. Cheng et al. (2024) employed a moderated parallel mediation model to examine the relationships between daily emotional experiences and smartphone addiction among college students, finding that stress served as a significant mediator and that gender moderated the protective pathway of positive emotions. Their finding that the stress-reduction route was especially pronounced among women resonates with the present observation that perceived stress differentially moderates the worship–cyberbullying link in a female-only sample, and underscores the importance of gender-sensitive analytical frameworks. Additionally, Jing et al. (2026) recently demonstrated that cyberbullying perpetration is associated with higher depressive symptoms among adolescents, with self-control and internet gaming addiction serving as partial mediators and meaning in life moderating the perpetration–self-control pathway. Collectively, these studies highlight the complex, multi-determined nature of cyberbullying and reinforce the necessity of examining both risk and protective factors within integrated theoretical frameworks that account for individual, relational, and contextual influences.

### Practical implications

The present findings yield several practical implications for intervention and prevention. At the individual level, young female adults should be encouraged to cultivate self-regulatory awareness and behavioral self-management strategies as fundamental safeguards against smartphone addiction. Education programs should aim to enhance awareness of the adverse consequences of excessive smartphone use, promote engagement in offline activities and outdoor pursuits, and foster active interpersonal communication. At the institutional level, universities should proactively monitor students’ smartphone use patterns and create enriching campus environments that provide meaningful alternatives to excessive screen time. Extracurricular activities, club organizations, and outdoor programs should be expanded to increase student engagement and reduce reliance on digital entertainment. Additionally, academic workloads should be calibrated to minimize stress-related vulnerability to maladaptive coping behaviors. At the family level, parents should maintain awareness of their children’s digital consumption patterns, provide guidance on normative celebrity admiration, and support the development of healthy peer relationships and interpersonal skills.

### Limitations and future directions

Several limitations of the present study should be acknowledged. First, the cross-sectional design precludes causal inference regarding the relationship between smartphone addiction and cyberbullying penetration. Although theoretical reasoning and prior evidence suggest that problematic Internet use precedes cyberbullying involvement, future research should employ experimental or longitudinal designs to establish temporal precedence and directional causality. Second, while the present study identified celebrity worship as a mediating mechanism, other potential mediators warrant investigation, including loneliness, empathy deficits, and moral disengagement. Moral disengagement, in particular, may attenuate the restraining influence of internalized moral standards, rendering individuals more susceptible to engaging in behaviors that violate their own ethical principles. Meta-analytic evidence has confirmed that moral disengagement positively predicts cyberbullying perpetration (Perren and Gutzwiller-Helfenfinger, 2012; Runions and Bak, 2015), and its association with reduced prosocial behavior and elevated antisocial behavior is well established (Bandura et al., 2001). Third, beyond perceived stress, additional moderators may qualify the smartphone addiction–cyberbullying pathway, including parenting style, self-esteem, and trait self-control. Future research should examine these potential boundary conditions to delineate the circumstances under which risk is most pronounced. Fourth, offering extra academic credit as an incentive may create undue influence, particularly among younger participants, potentially compromising the voluntariness of participation and introducing selection bias. Future studies should consider alternative recruitment strategies that minimize coercive incentives. Finally, the present study was conducted within an East Asian cultural context characterized by pronounced celebrity worship norms. Future research should examine the cross-cultural generality of the proposed model in diverse sociocultural settings.

## Conclusion

The present study examined the interplay among smartphone addiction, celebrity worship, perceived stress, and cyberbullying penetration in a sample of young female adults. Through empirical testing of a moderated mediation model, the findings demonstrate that smartphone addiction influences cyberbullying penetration both directly and indirectly through the mediating role of celebrity worship, and that perceived stress moderates the association between Borderline Pathological worship and cyberbullying penetration. These findings contribute to a more nuanced understanding of the psychological mechanisms underlying cyberbullying among young women and inform the development of targeted, multi-level intervention strategies aimed at reducing cyberbullying involvement in this vulnerable population.

## Data Availability

The raw data supporting the conclusions of this article will be made available by the authors, without undue reservation.

## Appendix

**Table A1:** The questionnaire in the present study (*N* = 525).

Variables	Items	Scale
Smartphone addiction	I get distracted by my smartphone while eating. I get distracted by my smartphone while studying. I frequently check my phone when talking with friends and family.	1 = completely disagree, 5 = completely agree
Celebrity worship (entertainment-social)	My friends and I enjoy discussing what my favorite celebrity has done. I like following my favorite celebrity because it brings me pleasure. When my favorite celebrity fails at something, I feel like a failure myself.	1 = completely disagree, 5 = completely agree
Celebrity worship (intense-personal)	I am fascinated by the personal details of my favorite celebrity’s life. When good things happen to my favorite celebrity, it feels as if they happen to me. I regard my favorite celebrity as my soulmate.	1 = completely disagree, 5 = completely agree
Celebrity worship (borderline pathological)	If I were lucky enough to meet my favorite celebrity and he/she asked me to do something illegal, I might do it. Hearing news about my favorite celebrity can relieve my current sadness. If someone gave me a large sum of money to spend freely, I would consider buying personal belongings once owned by my favorite celebrity.	1 = completely disagree, 5 = completely agree
Perceived stress	I worry about contracting illnesses. I have viewed a great deal of negative news. My academic progress has been disrupted. I worry about drifting apart from my classmates and friends.	1 = not at all stressed, 5 = very stressed
Cyberbullying penetration	I speak ill of other classmates or friends online or via text messages. I stir up conflicts between classmates or friends online or via text messages. I insult other classmates or friends online or via text messages. I threaten the personal safety of other classmates or friends online or via text messages.	1 = never, 5 = always
